# Referral patterns through the lens of health facility readiness to manage obstetric complications: national facility-based results from Ghana

**DOI:** 10.1186/s12978-019-0684-y

**Published:** 2019-02-18

**Authors:** Patricia E. Bailey, John Koku Awoonor-Williams, Victoria Lebrun, Emily Keyes, Mario Chen, Patrick Aboagye, Kavita Singh

**Affiliations:** 1 Independent consultant, Pittsboro, NC 27312 USA; 2 Policy Planning Monitoring and Evaluation Division, Ghana Health Service, Accra, Ghana; 3 FHI 360, 359 Blackwell Street, Suite 200, Durham, NC 27701 USA; 4 Family Health Division, Ghana Health Service, Accra, Ghana; 5 Maternal Child Health, MEASURE Evaluation/ Carolina Population Center, University of North Carolina at Chapel Hill, Chapel Hill, NC USA; 6 Department of Maternal and Child Health, Gillings School of Global Public Health, University of North Carolina at Chapel Hill, Chapel Hill, NC USA

**Keywords:** Referral system, Obstetrics, Emergency services, Maternal mortality, Ghana

## Abstract

**Introduction:**

Countries with high maternal and newborn mortality can benefit from national facility level data that describe intra-facility emergency referral patterns for major obstetric complications. This paper assesses the relationship between referral and facilities’ readiness to treat complications at each level of the health system in Ghana. We also investigate other facility characteristics associated with referral.

**Methods:**

The National Emergency Obstetric and Newborn Care Assessment 2010 provided aggregated information from 977 health facilities. Readiness was defined in a 2-step process: availability of a health worker who could provide life-saving interventions and a minimum package of drugs, supplies, and equipment to perform the interventions. The second step mapped interventions to major obstetric complications. We used descriptive statistics and simple linear regression.

**Results:**

Lower level facilities were likely to refer nearly all women with complications. District hospitals resolved almost two-thirds of all complicated cases, referring 9%. The most prevalent indications for referral were prolonged/obstructed labor and antepartum hemorrhage. Readiness to treat a complication was correlated with a reduction in referral for all complications except uterine rupture. Facility readiness was low: roughly 40% of hospitals and 10% of lower level facilities met the readiness threshold. Facilities referred fewer women when they had higher caseloads, more midwives, better infrastructure, and systems of communication and transport.

**Discussion:**

Understanding how deliveries and obstetric complications are distributed across the health system helps policy makers contextualize decisions about the pathways to providing maternity services. Improving conditions for referral (by increasing access to communication and transport systems) and the management of obstetric complications (increasing readiness) will enhance quality of care and make referral more effective and efficient.

## Plain English summary

Emergency referral is a strategy to improve access to life-saving services for women and their newborns during pregnancy, labor or shortly after birth. To be effective and efficient, receiving facilities must be ready to treat obstetric complications. In Ghana, as facility readiness levels increased, facilities tended to refer less. Readiness levels were highest at teaching and regional hospitals, followed by district hospitals. Findings suggest that most facilities were unable to adhere to national service delivery guidelines, which raises policy questions regarding where pregnant women, especially those with complications, should deliver and what quality improvement efforts are needed.

## Background

Emergency referral is critical to improving outcomes for time-sensitive conditions that underlie many unpredictable problems during pregnancy, delivery, and the postnatal period. This is especially true for poor, remote and rural populations where access to health services may be limited. Linking home to hospital, pre-hospital care, and timely treatment can improve maternal and neonatal survival.

How maternity services are organized, how they should be used and are used have been subjects of interest for decades. Where women ultimately give birth ranges widely from near universal hospital-based births to a mix of home births and institutional births at varying levels of care. The desired distribution of where institutional deliveries occur is a locally driven decision, influenced by policy, priorities, social norms and resources. Understanding how deliveries are spread across different types of facilities will inform how maternity services and the referral system should be designed and operationalized [1]. For example, if low risk births are highly centralized at a teaching hospital rather than across satellite clinics, the result can be inefficient use of scarce resources. However, when births are concentrated in health centers, the strategic location for emergency transport must be addressed.

Within the discourse on quality of care, childbirth services at the primary level (including health centers) is being challenged. Although governments often designate the provision of basic emergency obstetric and newborn care to health centers, the hard reality is few facilities fully function as such [2]. Evidence indicates that women are willing to travel farther and pay more for perceived higher quality services (respectful and competent health workers, available drugs and equipment, and access to more sophisticated services) [3]. Evidence also suggests that facilities with a higher volume of deliveries consistently measure higher quality of care and/or better maternal and newborn outcomes [4–6].

Structural and process components of quality of care help define the readiness of a facility to manage obstetric complications. Readiness to provide delivery and newborn care has been the focus of a limited set of studies [7, 8] and is also a measure of the obstacles to universal coverage and health system gaps [7, 9, 10].

Referral patterns described at a national level tell us how the health system is operationalized and how it is used. The degree to which health facilities and their staff are ready to respond to obstetric complications is key to providing good quality medical care. However, evidence of how these two components of service delivery interface is sparse.

The setting for this paper is the Ghanaian health system that follows a decentralized, three-tiered model. The primary level is made up of Community-based Health Planning and Services (CHPS) facilities, health centers, maternity homes and health clinics. At the primary level, district hospitals are considered the first referral level. The secondary level includes regional hospitals, and university teaching hospitals make up the tertiary level.

The Ghanaian health sector introduced several initiatives in the past 10–15 years designed to increase utilization of services and improve maternal and newborn indicators. In 2004 the National Ambulance Service was introduced and has expanded to at least 128 ambulance stations and can be found in all major cities, but rural and urban inequities persist [11, 12]. Many referred patients turn to taxis and local means of transport [12–14]. To increase health care utilization by pregnant women, the government also introduced a pro-poor initiative that exempted delivery fees in 2005, and extended the National Health Insurance Scheme (NHIS) to include antenatal care in 2008 [15, 16]. More poor women are using these services as a result of the NHIS [17], but it has not eliminated all out-of-pocket expenses [18], especially for drugs, ultrasound, and referral [19].

These initiatives have had mixed success. In 2015, the maternal mortality ratio in Ghana was 319/100,000 live births, unchanged from 325 in 2010; neonatal mortality declined from 31 per 1000 live births in 2010 to 27 in 2015 [20, 21]. The rate of institutional delivery increased from 67% in 2011 to 79% in 2017, while urban institutional delivery rates increased from 87 to 90% and rural rates from 53 to 68% [22, 23].

This paper aims to: 1) describe national referral patterns among women with obstetric complications and identify which complications are referred at each level of the health system; 2) assess health facility readiness to manage major obstetric complications and determine the extent to which readiness to treat aligns with the facility’s patterns of referral; and 3) identify other facility characteristics associated with referral.

## Methods

This is a secondary analysis of the National Emergency Obstetric and Newborn Care Assessment conducted by the Ghana Ministry of Health (MOH) and Ghana Health Service (GHS) in 2010. A detailed description of the assessment methods can be found elsewhere [24].

The assessment targeted public and private health facilities; the original selection criterion was based on the number of deliveries per month, where the minimum number eligible for inclusion ranged from 1 to 5 deliveries, depending on the geographic region. In the 3 Northern regions (Upper East, Upper West and Northern) one or more births per month was the cut off level while 5 was the cut off level for all other regions. A total of 1268 facilities were assessed, but in this paper, we selected only those facilities that had on average 5 or more deliveries per month based on the last 3 months before the survey, leaving 977 facilities in the sample.

The data collection instruments, used in more than 40 countries, were adapted to the Ghanaian health system [25]. Specific instruments covered facility infrastructure; availability of human resources; availability of drugs, equipment and supplies; performance of the emergency obstetric care signal functions; and retrospective service statistics. Service statistics included the number of women who delivered, number of women with obstetric complications, by type, and of these, the number of women referred to another facility. They also included the number of maternal deaths by cause of death. These tools were completed at all health facilities; tracer items were observed by data collectors but availability of most items was reported by facility staff. Data collectors reviewed service logbooks, labor and delivery registers, admissions and discharge records, and referral logbooks. They tallied events for the 12-month period April 2009 to March 2010 in the Upper East Region, and from July 2009 to June 2010 for the rest of the country. No other data sources were used.

We focused on complications that lead to the direct causes of maternal mortality: antepartum hemorrhage (placenta praevia or abruption), postpartum hemorrhage, retained placenta, severe pre-eclampsia and eclampsia, sepsis, prolonged/obstructed labor, ruptured uterus, ectopic pregnancy, and severe complications due to abortion (hemorrhage, infection, perforation, etc.). Women with multiple complications were counted only once; data collectors were trained to select the most life-threatening complication. Safe induced abortions were not included. Operational definitions of major direct obstetric complications were included in the data collectors’ manual and were drawn from *Monitoring emergency obstetric care: a handbook* [26].

### Main variables

Of primary interest was the extent to which facilities referred women experiencing a specific complication. We created a referral ratio where the numerator was based on the annual number of referrals out for each complication and the denominator was the annual number of women admitted with each complication. This ratio is an indicator of the level of referral occurring in each facility, though it is not a true proportion since the numerator was not always in the denominator because the data for each were sometimes gathered from different sources. Many Ghanaian facilities use referral registers, documenting whom they sent, when, where, and the indication for referral. Women who were admitted in labor or with a complication during pregnancy or postpartum, or developed a complication after admission were registered generally in labor and delivery logbooks. It was possible for a ratio to exceed 100% when the number of referrals for a complication exceeded the number of admissions with the same complication. For purposes of the regression analyses, we treated ratios that exceeded 100% as simply 100%.

We used a two-step process to classify whether each facility was ready to treat each of the complications of interest. First, a facility was defined as ready to provide each of the emergency obstetric care (EmOC) signal functions (newborn resuscitation with bag and mask was dropped given our focus on maternal complications) if it had both a health worker who could perform the signal function and the minimum package of health technologies (Table 1). Health worker performance was determined by a series of questions in the human resources module where we asked if each health worker category present at the facility provided each of the signal functions.

**Table 1:** Readiness to provide tracer drugs, supplies and equipment to perform the signal functions

Signal Function	Hospitals	Health Centers, Clinics, Maternity Homes, CHPS
Antibiotics (drugs in stock)	(Amoxicillin or ampicillin or ceftriaxone or chloramphenicol) and (metronidazole or clindamycin) and gentamicin	(Amoxicillin or ampicillin and gentamicin) OR (ceftriaxone and gentamicin) and (metronidazole or clindamycin)
Uterotonics (drugs in stock)	Has oxytocin or ergometrine (injection) and misoprostol	
Anticonvulsants (drugs in stock)	Has magnesium sulfate 50% or magnesium sulfate 20%	
Manual removal of placenta (supplies available)	Has examination gloves or long gloves	
Removal of retained products of conception (equipment functioning and available)	Has manual vacuum aspiration kit or dilation and curettage equipment	
Assisted vaginal delivery (equipment functioning and available)	Has vacuum extractor with different size cups or outlet forceps or other forceps.	
Obstetric surgery/ cesarean (equipment available and functioning)	Hospitals only Has anesthetic vaporizers and operating table and adjustable light and oxygen cylinders with manometer and flowmeter and (halothane/isoflurane or ketamine or lignocaine/lidocaine 2% or 1%)	
Blood transfusion (equipment and supplies available)	Has (reagents for blood typing/cross matching, and uses central blood supply, and has a functioning refrigerator for blood bank) or Has (reagents for blood typing/cross matching, and a functioning refrigerator for blood bank, empty blood bags, microscope, and blood tests for Hepatitis B, Hepatitis C, HIV, and syphilis) Note, if there was no answer to the question about central blood supply, we assumed they do not use it.	

Second, we mapped the eight signal functions to nine complications (Table 2), sometimes adjusting for facility type by assuming: 1) all hospitals had the potential to provide the comprehensive EmOC signal functions, and 2) health centers, health clinics, maternity homes and CHPS had the potential to provide the basic EmOC signal functions. For example, at hospital level, treatment of antepartum hemorrhage might entail the use of assisted vaginal delivery, surgery, and blood transfusion, whereas, at health center/clinic level, the only signal function expected would be assisted vaginal delivery. For two complications – ectopic pregnancy and uterine rupture – we assumed definitive treatment required surgery and thus no lower level facility could be considered ready to treat those complications.

**Table 2:** Mapping complications to signal functions

Complication	Signal functions for hospitals	Signal functions for health centers
Antepartum hemorrhage	Assisted vaginal delivery Blood transfusion Surgery (e.g. for placenta praevia)	Assisted vaginal delivery
Postpartum hemorrhage	Antibiotics Uterotonic drugs Manual removal of placenta Removal of retained products or fragments Blood transfusion Surgery (e.g. hysterectomy for ruptured uterus)	Antibiotics Uterotonic drugs Manual removal of placenta Removal of retained products or fragments
Retained placenta or fragments	Manual removal of placenta Antibiotics Uterotonics Blood transfusion	Manual removal of placenta Antibiotics Uterotonics
Pre-eclampsia/ eclampsia	Anticonvulsants Assisted vaginal delivery Surgery	Anticonvulsants Assisted vaginal delivery
Sepsis	Antibiotics Removal of retained products Surgery	Antibiotics Removal of retained products
Prolonged/ Obstructed labor	Uterotonics (for induction) Assisted vaginal delivery Surgery (e.g. cesarean)	Uterotonics (for induction) Assisted vaginal delivery
Ruptured uterus	Antibiotics Surgery (e.g. hysterectomy or uterine repair) Blood transfusion	Assumption: refer all cases
Complications of abortion	Antibiotics Uterotonics Removal of retained products Blood transfusion Surgery	Antibiotics Uterotonics Removal of retained products
Ectopic pregnancy	Blood transfusion Surgery	Assumption: refer all cases

Readiness to treat complications and readiness to provide the signal functions were informed by the Ghana *National Safe Motherhood Service Protocol* that specified care at community level, health center level and first referral or district level care [27].

### Statistical analysis

The descriptive results are reported as means, ranges, ratios, and percentages. We used simple linear regression of the referral ratio. All analyses were performed using STATA v. 13 software [28]. Statistical tests were shown only for the regression results, with significance measured at the 0.05 level.

### Ethical statement

No patient or staff names or other personal identifiers were recorded on the data collection instruments and were never part of the electronic database. Access to the facilities was facilitated with a letter from GHS and medical directors granted data collectors permission to talk to staff and view facility records. The Navrongo Health Research Centre in Ghana provided ethical approval.

## Results

### National patterns of institutional delivery, maternal mortality and referral among women with major direct obstetric complications

Among the 977 facilities, the assessment documented 428,129 deliveries, 66% of which occurred in hospitals (52% in district hospitals) (Table 3). The remaining 34% took place at health centers, clinics, maternity homes, and CHPS compounds. Among the deliveries, 38,142 (9%) women had a major direct obstetric complication as listed in Table 3; an additional 14,208 women (3%) had “other direct obstetric complications.” Among the 38,142 women, however, 486 women died. The institutional maternal mortality ratio based on the 9 common obstetric complications in Table 3 was 274/100,000 deliveries at teaching and regional hospitals compared to 137 at district hospitals. Very few maternal deaths occurred in lower level facilities.

**Table 3:** Number of deliveries, maternal deaths, obstetric complications and referrals by complication type and facility type

	National total *n* = 977	Teaching & Regional hospitals *n* = 12	District hospitals *n* = 248	Health centers *n* = 439	Health clinics *n* = 113	Maternity homes *n* = 129	CHPS compounds *n* = 36
Deliveries n (%)	428,129	59,838	223,376	96,950	18,614	25,850	3501
	(100%)	(14%)	(52%)	(23%)	(4%)	(6%)	(1%)
Total deliveries with direct obstetric complications^a^							
Admitted	38,142	9418	24,518	2932	537	644	93
Deaths^b^	486	164	307	13	2	0	0
Referred n (%)	8009 (21%)	66 (1%)	2097 (9%)	4292 (146%)	601 (112%)	858 (133%)	95 (102%)
Antepartum hemorrhage							
Admitted	3921	1191	2147	360	134	81	8
Deaths	33	6	26	1	0	0	0
Referred	1110 (28%)	0	277 (13%)	597 (166%)	115 (86%)	112 (138%)	9 (112%)
Postpartum hemorrhage							
Admitted	3800	625	2226	629	163	127	30
Deaths	111	45	61	4	1	0	0
Referred	852 (22%)	9 (1%)	159 (7%)	454 (72%)	99 (61%)	117 (92%)	14 (47%)
Retained placenta							
Admitted	2312	404	1527	279	33	66	3
Deaths	10	0	8	1	1	0	0
Referred	512 (22%)	0	139 (10%)	272 (97%)	39 (118%)	58 (88%)	4 (133%)
Pre-eclampsia/eclampsia							
Admitted	3496	1638	1718	95	15	25	5
Deaths	137	52	84	1	0	0	0
Referred	422 (12%)	1 (< 1%)	186 (11%)	172 (181%)	21 (140%)	38 (152%)	4 (80%)
Sepsis							
Admitted	489	120	302	37	12	18	0
Deaths	60	25	35	0	0	0	0
Referred	66 (13%)	0	24 (8%)	27 (73%)	5 (42%)	10 (55%)	0
Obstructed/ prolonged labor							
Admitted	15,489	2912	10,898	1208	132	297	42
Deaths	14	2	11	1	0	0	0
Referred	4220 (27%)	53 (2%)	1066 (10%)	2298 (190%)	256 (194%)	491 (165%)	56 (133%)
Uterine rupture							
Admitted	440	134	304	2	0	0	0
Deaths	36	11	25	0	0	0	0
Referred	28 (6%)	0	25 (8%)	3 (150%)	0	0	0
Ectopic pregnancy							
Admitted	2176	983	1170	11	8	4	0
Deaths	15	6	9	0	0	0	0
Referred	117 (5%)	3 (< 1%)	79 (7%)	22 (200%)	6 (75%)	7 (175%)	0
Abortion complications							
Admitted	6019	1411	4226	311	40	26	5
Deaths	70	17	48	5	0	0	0
Referred	682 (11%)	0	142 (3%)	447 (144%)	60 (150%)	25 (96%)	8 (160%)

^a^Total deliveries with direct obstetric complications do not include 14,208 women who fell into the category of “other direct obstetric complications”

^b^Institutional direct maternal deaths do not include 107 due to “other direct causes”

**Fig. 1:**
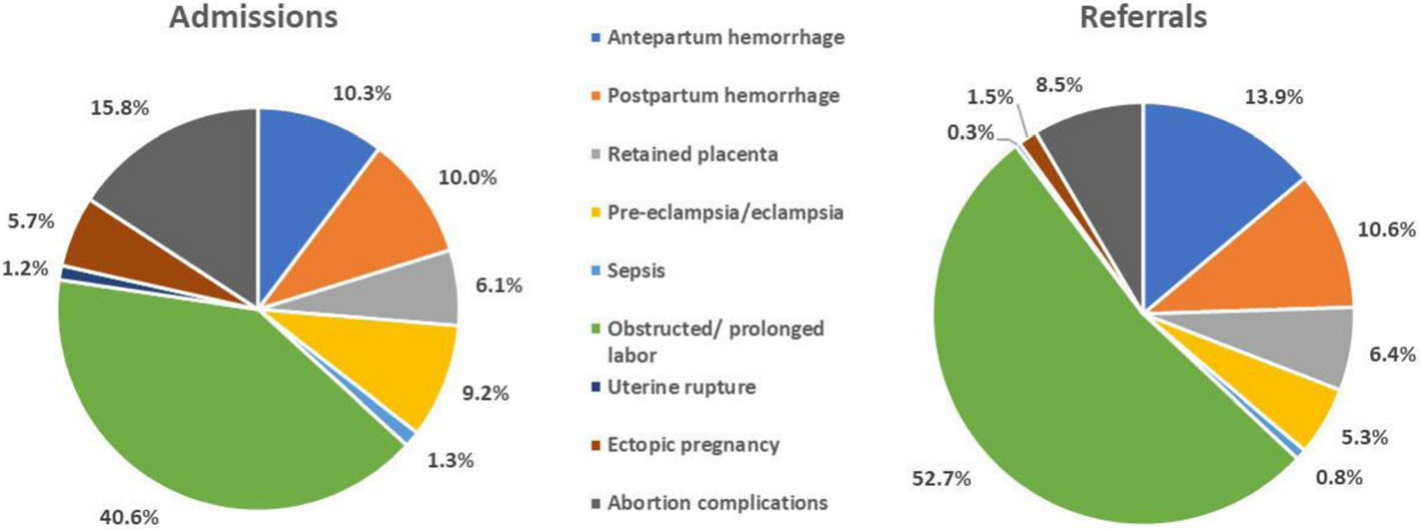
Distribution of admissions with direct obstetric complications (*n* = 38,142) and women referred (*n* = 8009) according to type of complication

Among women with complications, 8009 (21%) were referred. The leading complications among admissions were prolonged/obstructed labor (41%) and abortion-related complications (16%) (Fig. 1). The leading referral indications were prolonged/obstructed labor (53%) and antepartum hemorrhage (APH) (14%).

Teaching and regional hospitals, the apex of the referral hierarchy, referred out fewer than 1% of the women with a major obstetric complication (Table 3). On these occasions, usually a regional hospital refers to a teaching hospital. District hospitals played a prominent role in the management of obstetric complications by receiving 24,518 women with complications and referring 9% of them. In contrast, lower level facilities relied heavily on referral.

Referral patterns varied according to obstetric complication, providing some indication of which complications were treated at each level of the health system. Proportionately, district hospitals referred more women with APH (13%) than they did women with abortion complications (3%).

The referral ratio approached or exceeded 100% for most obstetric complications at lower level facilities. The two exceptions were some cases of postpartum hemorrhage (PPH) and sepsis that presumably were managed on site. Only six of the eight cases of ectopic pregnancy at health clinics were referred, likely the result of under-reporting referrals since the facilities in question had no surgical capacity to manage ectopic pregnancy and they reported no maternal deaths. Lower level facilities documented admitting (or referring) few cases of uterine rupture or ectopic pregnancy, suggesting that these women might have bypassed lower level facilities to go directly to a hospital, or the diagnosis (recorded complication) was incomplete or in error.

### Facility readiness to provide EmOC signal functions and treat major direct obstetric complications

**Fig. 2:**
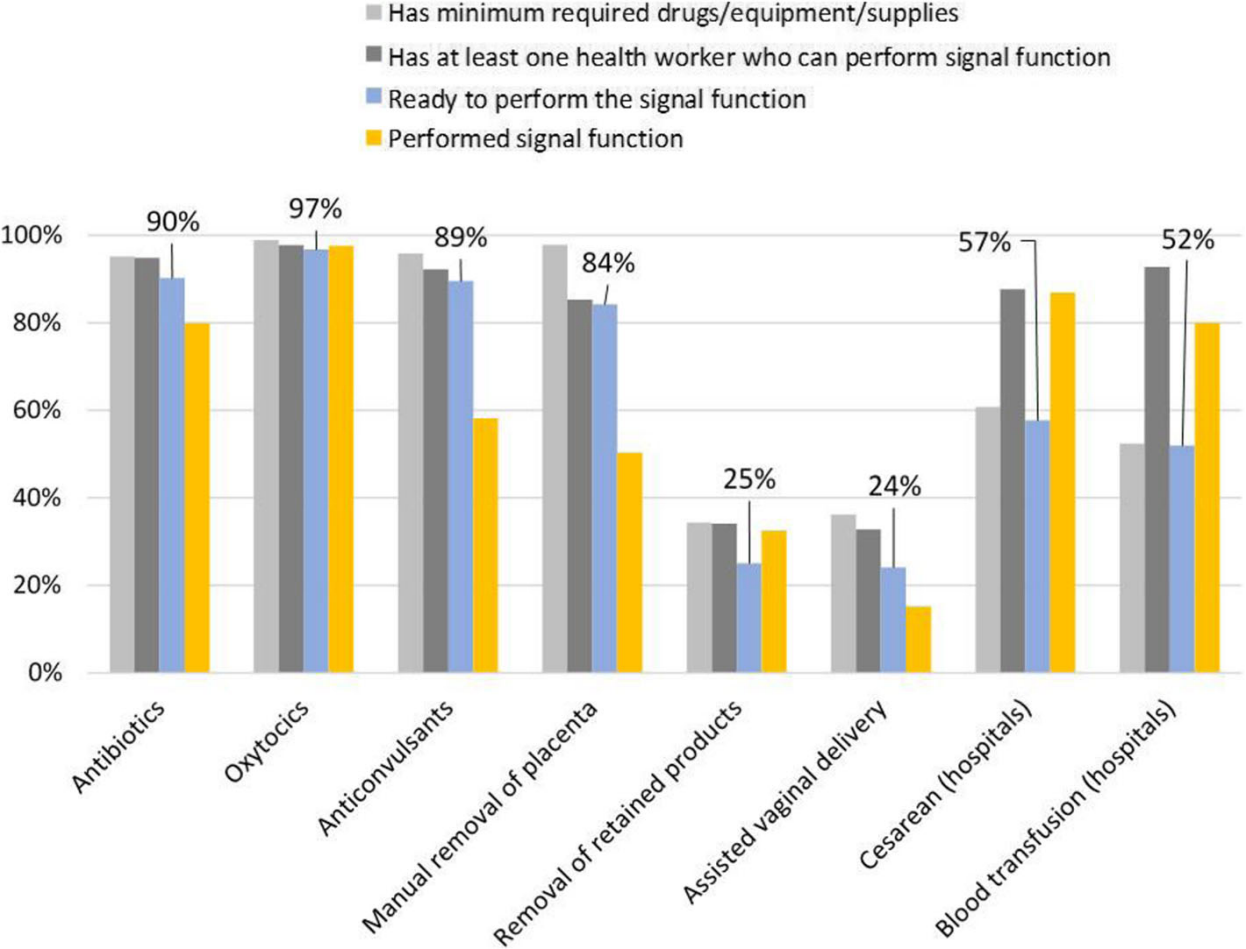
Readiness to provide the emergency obstetric signal functions

High proportions of facilities (> 84%) were ready to perform intramuscular or intravenous antibiotics, uterotonics, anticonvulsants or manual removal of placenta, while relatively few facilities (24%) were ready to perform removal of retained products or assisted vaginal delivery (Fig. 2). In the latter two cases, both human resources and the equipment and supplies needed to perform the interventions were missing at similar rates.

Readiness to perform cesarean delivery and blood transfusion was assessed only for hospitals since lower level facilities generally do not offer those services. Some hospitals lacked the minimum required items for surgery and blood. Yet, performance of these two signal functions in the last three months exceeded readiness, suggesting that these life-saving services took place in suboptimal conditions.

**Fig. 3:**
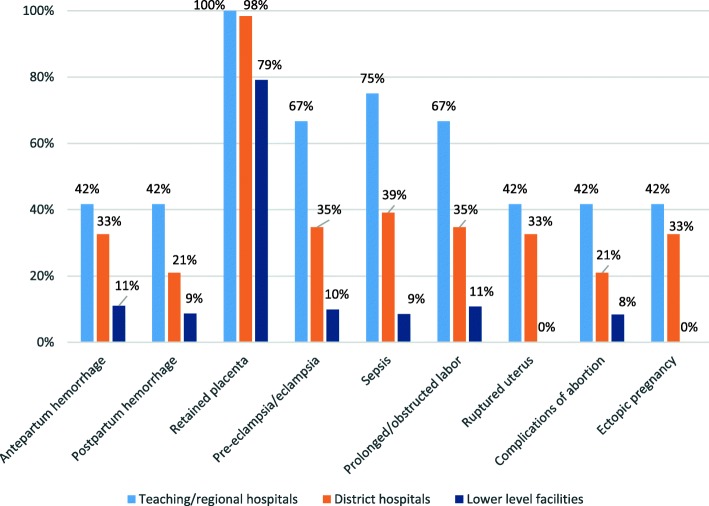
Readiness to treat complications by facility type

Readiness to treat complications was low at all levels (Fig. 3), the exception being retained placenta. Teaching and regional hospitals were better prepared than district hospitals; the biggest differentials were observed for postpartum hemorrhage and complications of abortion, where 42% of teaching and regional hospitals were ready compared to only 21% of district hospitals. Roughly 10% of lower level facilities were ready to treat these same complications, again apart from retained placenta. No lower level facilities were ready to treat uterine rupture or ectopic pregnancy, since both required surgical care.

Readiness to treat complications varied by specific facility type (Table 4). The pattern was unambiguous: the 12 teaching and regional hospitals consistently demonstrated the greatest readiness, followed by district hospitals, maternity homes, health clinics, health centers and CHPS.

**Table 4:** Number and percentage of facilities ready to treat complications, by facility type

Complication	Teaching + Regional Hospitals (*n* = 12)		District Hospitals (*n* = 248)		Health centers (*n* = 439)		Health clinics (*n* = 113)		Maternity homes (*n* = 129)		CHPS (*n* = 36)	
	n	%	n	%	n	%	n	%	n	%	n	%
Antepartum hemorrhage	5	41.7	81	32.7	31	7.1	13	11.5	35	27.1	0	0.0
Postpartum hemorrhage	5	41.7	52	21.0	21	4.8	12	10.6	28	21.7	1	2.8
Retained placenta	12	100.0	244	98.4	340	77.4	99	87.6	106	82.2	22	61.1
Pre-eclampsia/eclampsia	8	66.7	86	34.7	29	6.6	13	11.5	29	22.5	0	0.0
Sepsis	9	75.0	97	39.1	18	4.1	14	12.4	29	22.5	0	0.0
Prolonged/obstructed labor	8	66.7	86	34.7	30	6.8	13	11.5	34	26.4	0	0.0
Ruptured uterus	5	41.7	81	32.7	0	0.0	0	0.0	0	0.0	0	0.0
Complications of abortion	5	41.7	52	21.0	18	4.1	13	11.5	29	22.5	0	0.0
Ectopic pregnancy	5	41.7	81	32.7	0	0.0	0	0.0	0	0.0	0	0.0

To determine whether readiness to treat an obstetric complication aligned with the frequency that a facility referred women with that complication, we conducted a simple linear regression of the referral ratio for each complication, as predicted by facility readiness to treat each complication (Table 5). For example, a facility that was ready to treat APH was predicted to have a 0.36 smaller referral ratio for APH than facilities that were not ready to treat APH on average. Greater readiness translated into significantly less referral for every complication except uterine rupture. All hospitals and lower level facilities were included in this analysis.

**Table 5:** Linear regression of how facility readiness to treat major obstetric complications aligns with referral

Complication	Estimate	*p*-value
Antepartum hemorrhage	−0.36371	< 0.001
Postpartum hemorrhage	−0.2031	< 0.001
Retained placenta	−0.38635	< 0.001
Pre-eclampsia/eclampsia	−0.32708	< 0.001
Sepsis	−0.30346	< 0.001
Obstructed/ prolonged labor	−0.30236	< 0.001
Uterine rupture	−0.10086	0.111
Abortion complications	−0.25748	< 0.001
Ectopic pregnancy	−0.17525	0.006

### Additional facility characteristics associated with referral

Other facility characteristics also drove referral. We looked at four levels of referral frequency (Table 6):

1. facilities that reported no women with complications and no referrals (*n* = 167);
2. facilities that referred more than half of women with complications (*n* = 534);
3. facilities that referred between 10 and 50% (*n* = 81); and
4. facilities that referred fewer than 10% (*n* = 195).

**Table 6:** Percent distribution, percent and mean number of facilities by magnitude of referral out according to facility characteristics

	Reported no women with complications and no referrals (*n* = 167)	Reported referring women with complications		
		Referred > 50%^a^(*n* = 534)	Referred 10–50% (*n* = 81)	Referred fewer < 10% (*n* = 195)
Facility type				
Teaching & regional hospitals	0.0	0.0	1.2	5.6
District hospitals	15.0	7.9	48.2	72.8
Health centers	45.5	57.9	33.3	13.9
Health clinics	14.4	14.4	4.9	4.1
Maternity homes	16.8	16.5	9.9	2.6
CHPS compound	8.4	3.4	2.5	1.0
Managing authority				
Public / government	62.9	66.3	70.4	48.2
Private for-profit	31.7	19.7	19.8	27.7
Private not-for-profit	5.4	14.0	9.9	24.1
Has electricity	90.4	94.4	92.6	98.0
Has source of water	68.3	70.9	75.3	89.7
Has mode of motorized transport	43.1	45.9	64.2	73.9
Has means of communication	32.3	35.4	55.6	80.5
Volume of deliveries				
Monthly mean	13.6	20.4	46.2	96.3
Monthly range	3–96	3–286	4–259	3–1107
Mean EmOC readiness score^b^	3.8	3.9	5.0	6.0
Mean number of signal functions performed in last 3 months	3.5	3.7	5.7	6.9
Midwives on staff (*n* = 874)				
None	68.8	65.9	42.9	14.9
1	19.2	23.4	26.0	22.1
2–4	11.4	9.7	27.3	43.7
5 or more	0.7	1.1	3.9	19.3
Has operating theater^c^	14.2	6.2	48.2	79.0

^a^Includes facilities that did not officially admit women with complications but had evidence of referring women

^b^EmOC readiness score is a composite measure of signal function readiness, where each SF is worth 1 point

^c^First column is based on *n* = 162; second column is based on *n* = 520

The first category consisted largely of small volume facilities that conducted 14 deliveries on average per month. Eighty-five percent were lower level facilities. Compared with the other groups, they had the weakest infrastructure as measured by the presence of electricity or water, on-site communication (existence of landline, cell phone or radio owned by the facility) or transportation. These facilities had the lowest EmOC signal function readiness score and performed an average of 3.5 signal functions in the last 3 months, which indicates that some of these facilities did treat or stabilize women with complications, contrary to the fact that this group registered no women with complications and no referrals. Two-thirds had no midwife on-site, but 14% had an operating theater (22 were district hospitals and one a maternity home), one-quarter of which tallied more than 100 deliveries in the last three months. Some of these facilities either had very poor records or data collectors failed to access the information.

The second group of facilities were predominantly lower level facilities with fewer district hospitals or operating theatres than the first group. Otherwise, they showed marginal increases over the first group.

The third group consisted largely of district hospitals with operating theatres. The volume of deliveries was more than twice (46 births per month) that of the second group, about two-thirds had their own transport, and on average they performed almost 6 signal functions, and had an EmOC readiness score of 5.

The final group consisted predominantly of hospitals and demonstrated the most robust infrastructure, the highest birth loads, readiness scores, and signal function performance.

## Discussion

Among women delivering in a health facility in Ghana, most used hospitals, especially district hospitals, despite 45% of the population living in rural areas [29]. Prolonged/obstructed labor was the most prevalent obstetric complication reported and also the leading indication for referral. Lower level facilities tended to refer most, if not all, women with major obstetric complications, the exceptions were PPH and sepsis. Health centers, for example, appear to have resolved about 25% of cases of PPH and sepsis.

Readiness to treat complications predicted a lower likelihood of referral. Health facilities tended to refer less as their infrastructure improved, delivery caseload increased, the number of midwives increased, and EmOC signal function readiness and performance increased. Facilities that relied most heavily on referral were the least likely to report on-site transport or a means of communication, critical components of a referral system.

District hospitals are the backbone of the Ghanaian system, with 20 district hospitals for every teaching or regional hospital; they are more accessible to the population and delivered 52% of all institutional births. District hospitals also treated almost two-thirds of all obstetric complications (and probably more since 22 district hospitals had missing data), and referred fewer than 1 in 10 women with complications. Despite their critical role in the health system, readiness to treat PPH and complications of abortion, for example, existed in only 21% of the district hospitals. Thus, staff worked in suboptimal conditions, with incomplete equipment, lacked drugs recommended by the protocols, making do with what they had. Fewer than 60% of all hospitals were considered ready to provide cesarean surgery or blood transfusions, but in each case, more than 80% of these same facilities had provided the service in recent months. The disparity in maternal mortality between the teaching/regional hospitals and district hospitals might be an indication that the former treated the women with the most severe complications, some of whom surely reached the top of the referral chain in precarious conditions. Given the large demand for life-saving services at all hospitals, gaps in readiness must be targeted for additional resources.

Centralizing complication management at hospitals raises questions about the role that basic emergency obstetric care plays or could play at mid-level facilities. Building the capacity to provide basic emergency care assumes training and equipping (often mid-level) providers at subdistrict level, potentially making referral more effective, more efficient and in some cases obviate the need for referral [30]. Treatment of abortion complications is an example of how decentralizing complication management could benefit women and decongest hospitals. Numerous high-, middle- and low-income countries including Ghana have demonstrated that mid-level professionals in non-hospital environments can safely and effectively treat abortion-related complications [31–33]. Yet we found that lower level facilities treated few women with severe abortion complications, relying instead on referral. Only 24% of all 977 facilities had the required equipment and health worker to remove retained products of conception. The *National Safe Motherhood Service Protocol* clearly states that pregnancies less than 12-weeks gestation, with signs of inevitable or incomplete abortions, can be managed at health centers. We also looked at women treated for less severe abortion complications, and again most cases (94%) received post-abortion care in hospitals. Similarly, 85% of legal terminations of pregnancy were performed in hospitals (data not shown). The gaps in equipment and staff to provide post-abortion care at lower levels may be related to the stigma or politics that surround abortion as discussed in several recent studies with health care providers [31, 34].

In our study, the transfer of women from one facility to another took place in poorly prepared environments where most obstetric complications could not be adequately treated. For most obstetric complications, fewer than 40% of all hospitals and 10% of lower level facilities were considered ready to treat those complications. Thus, the strategy to refer probably made good medical sense to health workers, especially at lower levels, but referral also presents women and families with serious access problems related to transport, financial burdens, and other inconveniences.

The extent of the readiness gaps to manage complications as measured against standardized national protocols for stabilization, definitive treatment and referral was a major finding of this study, and could be used as a roadmap for improving the quality of care. A substantial readiness gap was noted across all levels of the health system, even among the 12 teaching and regional hospitals, the top of the referral chain, where only 5 were deemed ready to treat APH, PPH, ruptured uterus, ectopic pregnancy, and complications of abortion. Deaths due to these five causes accounted for almost half of the institutional maternal deaths.

A strength of this paper is that we captured an entire country’s referral hierarchy, estimated how many pregnant or recently pregnant women were referred, and identified the indications for referral at each level of the system. Furthermore, we gleaned insight into structural and process components of delivering acute care services that helped reveal the complexity of referral. The methods we used to measure readiness to perform the signal functions, and subsequently readiness to treat complications, may be overly restrictive and could be refined.

The EmONC assessment was not designed to primarily study referral and therefore many topics of interest were not included. For example, we were unable to measure individuals’ continuous trajectory of care seeking, complication severity and appropriateness of referral or even outcomes to referral. Data collectors did not review patient charts for the 8000+ women admitted with obstetric complications. In our case, all referrals occurred between facilities where the judgement to refer was made by health workers, but surely some referrals were unnecessary or avoidable while others should have been made. Other Ghanaian researchers have observed excessive referral in studies based on selected facilities, especially from lower levels. One study observed a relatively high proportion of referrals among women in labor whose membranes were still intact and might have been managed with artificial rupture [34]; another noted that health centers were poorly equipped, and staff chose to refer rather than to treat in suboptimal conditions [35].

The referral data were limited to referrals out and we could not estimate compliance with referral advice. Nor do we know where women went when referred, thus, we could not determine to what extent the overall number of women with complications was over-estimated by double-counting at the facility where a woman first sought treatment and again at the referral destination. The referral ratio was calculated at the facility level, and thus over-counting in those analyses was not a concern.

We believe that under-counting obstetric complications and referrals may have been more serious than over-counting. Based on Table 6 (first column), we provided evidence from 22 district hospitals that contributed no women with complications and no referrals due to incomplete recording in the primary sources or by data collectors at the time of data collection. Referral ratios that exceeded 100% were further evidence of inconsistent practices regarding the recording of admissions and of referrals. This is a data quality issue that should be addressed with the appropriate support and strengthening of the information system with, for example, referral tracking. We were also surprised that APH was the second most prevalent indication for referral since previous studies have indicated that hypertensive diseases in pregnancy were the second most common indication for referral [36]. We cannot rule out that some incomplete abortions might have been misclassified as antepartum hemorrhage.

## Conclusions

This ecological study highlights several important health system findings: maternity service delivery appeared to be skewed towards the district hospital, facility readiness to manage complications was generally low but varied by type of facility, and subdistrict facilities, the least ready, tended to refer almost all women with complications. These lower level facilities were also the least likely to have formalized communication or transportation systems.

Although this study flags the de facto policy of lower level facilities referring almost all women with complications, we cannot say that health workers referred too much or too little. However, in an environment where the financial and cultural costs of referral are considerable, excessive use of services is probably not driving the dynamics of referral. An element of the decision-making process for referral is judgment, and health workers should not be discouraged from referring. The consequences of over-referral can and should be managed, but under-referring or delaying referral can lead to fatal consequences, especially if referral hospitals are ill-prepared.

## Abbreviations

APH: Antepartum hemorrhage
CHPS: Community-based Health Planning and Services
EmOC: Emergency obstetric care
GHS: Ghana Health Service
MOH: Ministry of Health
NHIS: National Health Insurance Scheme
PPH: Postpartum hemorrhage
